# COVID‐19: From pathogenesis models to the first drug trials

**DOI:** 10.1111/1751-7915.13611

**Published:** 2020-06-23

**Authors:** Harald Brüssow

**Affiliations:** ^1^ Department of Biosystems Laboratory of Gene Technology KU Leuven Leuven Belgium

## Abstract

The number of people infected with SARS‐CoV‐2, and sadly dying from COVID‐19, has exploded, and so the amount of literature on the novel coronavirus and the disease it causes has increased proportionately. The case numbers in some countries are beyond the epidemic peak, but the uncertainty about a second wave keeps politicians and societies under pressure. Appropriate decision‐making and winning support from the population depends on precise scientific information rather than leaving the field to scaremongers of all proveniences. This mini‐review is an update of earlier reports (Brüssow, *Microb Biotechnol* 2020a;13:607; Brüssow, *Microb Biotechnol* 2020b; https://doi.org/10.1111/1751-7915.13592).

## Autopsies

Autopsies can provide valuable insight into new diseases which complement or correct observations made by clinicians. Surprisingly, it took a while until the first pathology reports were published for COVID‐19 patients. This delay is explained by the emergency situation of a pandemic, the limited number of complete sections that can be conducted by a pathology institute, biosafety issues and a decreasing role of pathology in medical research.

In a US COVID‐19 patient, the lungs were of firm consistency and heavy with oedema. Upon histological analysis, diffuse alveolar damage (DAD) was diagnosed. Thrombi were seen in small lung arteries, and there was congestion in capillaries. Alveoli showed hyaline membrane formation. The lung tissue displayed chronic inflammation with invasion of T lymphocytes. The patient died of cardiac arrest (Barton *et al*., 2020).

A Chinese COVID‐19 patient showed, likewise: DAD, hyaline membranes and pulmonary oedema together with inflammatory infiltrates and multinuclear syncytia, typical cytopathic effects of coronaviruses. The patient showed overactive T cells with a marked increase in Th17 cells and high cytotoxicity of CD8 T cells, potentially explaining the severe immune injury seen in the lung of this patient (Xu *et al*., 2020).

The first larger series of autopsies was published for 12 German COVID‐19 patients. Their average age was 73 years, and most were obese. Deep venous thrombosis was found in 7 patients that had not been suspected before death. Pulmonary embolism was the cause of death in 4 patients. The thrombi were derived from deep veins in the lower extremities. In all cases, the cause of death was found in the lungs which were congested and heavy, weighing 2 kg instead of 800 g. At a histological level, diffuse alveolar damage was seen in 8 patients. Viral RNA was detected with high titres in liver, heart and kidney. Clinical chemistry showed increased levels of lactate dehydrogenase, D‐dimer and C‐reactive protein, suggesting coagulopathy as a complication in these severe COVID‐19 cases (Wichmann *et al*., 2020). Interestingly, obesity is increasingly seen as a risk factor for severe disease. In a retrospective study with 3615 Covid‐19 patients from New York City, clinicians had described obesity as a risk factor for intensive care need (OR 1.8 to 3.6) in patients < 60 years (Lighter *et al*., 2020).

## Pathogenesis

### Coagulopathy

Clinicians had already elaborated concepts for COVID‐19 pathogenesis that concur with these observations from the autopsies. In one model, COVID‐19 patients show a diffuse pulmonary intravascular coagulopathy which is – in contrast to disseminated intravascular coagulation (DIC) – restricted to the lung vascular bed. The increased D‐dimer concentrations reflect the pulmonary thrombosis followed by fibrinolysis, and the elevated cardiac enzymes reflect the stress imposed on the heart ventricle by pulmonary hypertension. According to this model, it is not the viral infection but the vascular thrombosis induced by the infection which underlies the severe pathology. The coagulopathy might be caused by an activation of an immune mechanism involving macrophages as observed in ‘macrophage activation syndrome’ (MAS), suggesting targets for drug intervention (McGonagle *et al*., 2020).

### Cytokine storm

Another popular hypothesis for severe COVID‐19 pathology is based on observations of acute respiratory distress syndrome (ARDS) where pneumonia, sepsis or aspiration pneumonia lead via a release of pro‐inflammatory cytokines from immune cells (‘cytokine storm’) to severe lung damage. In COVID‐19, the recruitment of the cellular virus receptor by the infecting virus causes disappearance of the angiotensin‐converting enzyme‐2 (ACE‐2) from the cell surface and by its absence results in an increase in unprocessed angiotensin 2 (Ang II). Via a cascade of reactions involving the metalloproteinase ADAM17, increased Ang II concentrations induce the cytokines tumour necrosis factor alpha (TNF‐α) and interleukin 6 (IL‐6) which activate the IL‐6 amplifier (IL‐6 AMP), leading to the release of inflammatory cytokines. In parallel, the virus induces inflammatory cytokines by activating the NF‐κB pathway via pattern recognition receptors (PRRs). This hypothesis explains much of the observed pathology in COVID‐19 and suggests a number of targets for pharmacological intervention (Hirano and Murakami, 2020). The expression of immune genes was studied in 3 COVID‐19 patients with severe, moderate and mild disease. Elevated plasma levels of pro‐inflammatory cytokines (IL‐1β, IL‐8 and TNF‐α) were observed, although they occurred after the peak of respiratory failure (Mehta *et al*., 2020; Ong *et al*., 2020).

### Viral sepsis

Only 5% of COVID‐19 cases show severe infections that lead to death in about 1.4 % of cases from severe lung injury and multiorgan dysfunction. Chinese clinicians observed that critically ill patients showed signs of shock with cold extremities, weak peripheral pulse, metabolic acidosis and microcirculation dysfunction. This combination of symptoms suggests septic shock, but in 76% of these patients, SARS‐CoV‐2 was the only pathogen. These researchers propose ‘viral sepsis’ as the cause for severe COVID‐19, where the virus infects the lymphocytes, induces detrimental immune reactions and infects vascular epithelia leading to disseminated intravascular coagulation (Li *et al*., 2020a).

These models of COVID‐19 pathogenesis are not mutually exclusive. SARS‐CoV‐2 infections have a variable presentation ranging from causing: no symptoms; only mild symptoms; or severe disease necessitating hospitalization, if not intensive care. In addition, the clinical presentation differs with severe COVID‐19 depending whether diffuse alveolar damage, coagulopathy, cytokine overproduction or viral sepsis dominates the disease.

### Patient data

Viral sepsis and cytokine storm might, in fact, be connected. Greek researchers compared immune activation and dysregulation in patients with pneumonia caused by bacteria, influenza virus or SARS‐CoV‐2. At the moment of hospitalization, COVID‐19 patients were clinically less affected than patients with bacterial pneumonia. However, a common observation in COVID‐19 patients is that one week after hospitalization they progressed from a relatively good clinical state into sudden deterioration. These researchers observed low expression of the human leucocyte antigen (HLA)‐DR on CD14 monocytes in COVID‐19 patients in need of mechanical ventilation. The patients showed a unique combination of defective antigen presentation and lymphopenia. Interleukin‐6 (IL‐6) and C‐reactive protein (CRP) were significantly increased in severe cases. IL‐6 is known to inhibit HLA‐DR expression. An inverse correlation between these markers was, in fact, observed in severe cases. The authors of this study suggest a clinical trial exploring a specific blocker of the IL‐6 pathway such as tocilizumab to restore the expression of HLA‐DR, and to alleviate the immune‐paralysis seen in severe COVID‐19 cases. (Giamarellos‐Bourboulis *et al*., 2020) Data from Wuhan concur with these observations. When investigating 48 COVID‐19 patients with distinct disease severity, the clinicians observed viral RNA in the blood (‘RNAaemia’) of five critically ill patients, two of whom died of respiratory failure. Interestingly, all 5 patients also showed sharply increased IL‐6 levels. Viral RNA in the blood and high IL‐6 were biomarkers of severe disease (Chen *et al*., 2020b).

## Molecular biology approaches

### Antiviral response in cells, ferrets and patients

Defence against viral infection begins at the cellular level. Cells detect a replicating virus by pattern recognition receptors that signal the presence of unusual RNA structures. These receptors, when bound to these aberrant RNA molecules, activate the transcription factors IRF and NF‐κB, which launch two antiviral programmes: (i) induction of type I and III interferon (INF‐I, INF‐III), which upregulate interferon‐stimulated genes (ISG); (ii) recruitment of specific leucocytes by chemokine secretion. US researchers studied the viral and cellular transcriptional response upon infection of cell cultures and in animal models with different respiratory viruses including influenza A virus and SARS‐CoV‐2. In cell culture, viral RNA represented 50% or more of all cellular messenger RNA (mRNA). The external addition of INF‐I reduced SARS‐CoV‐2 replication dramatically. However, infection of cells with SARS‐CoV‐2 did not induce an interferon response, but a strong chemotactic and inflammatory response instead. In nasal washes from infected ferrets, viral RNA represented < 2% of all mRNA. SARS‐CoV‐2 induced a different cellular transcription response compared to the influenza virus: a cytokine response was followed by gene transcripts of cell death and leucocyte activation. Postmortem lung samples from COVID‐19 patients showed no INF‐I and INF‐III, but a robust chemokine transcription instead. The observations were confirmed in serum samples of COVID‐19 patients compared with samples from control subjects, showing no interferon, but a cytokine response. The data suggest that SARS‐CoV‐2 has learned to suppress interferon induction and to disarray the antiviral response (Blanco‐Melo *et al*., 2020).

### ACE‐2 expression data

Another impressive US study, so far only available as a preprint, investigated the expression of ACE‐2, the viral receptor of SARS‐CoV‐2, and TMPRSS2, the protease activating viral fusion with cell membranes leading to cell entry, in a large set of 695 healthy and asthmatic children from Puerto Rico (Gala II study). ACE‐2 expression was highly correlated with the expression of a gene network associated with cytotoxic T cells, induced in virally infected epithelia and correlated with a network of interferon and epithelial viral response genes. Notably, 78% of subjects showing high interferon levels were asymptomatic respiratory virus carriers (not with SARS‐CoV‐2, the study preceded the epidemic) compared with 10% asymptomatic infections in subjects displaying only low interferon levels. Eighteen children were infected with seasonal coronaviruses, and they demonstrated an increased ACE‐2 expression. The researchers correlated nasal ACE‐2 and TMPRSS2 gene expression with genome wide genetic variation data for these children. They identified a variant locus downstream of the transcription start site of the ACE‐2 gene which was associated with a large decrease in ACE‐2 expression. They also found variants that were associated with increased or decreased TMPRSS2 expression. The distribution of these variants showed clear differences in diverse populations worldwide. It will be interesting to study whether distinct expression pattern and the observed genetic variants explain the strikingly different clinical symptomatology between individuals and whether these genetic markers can explain geographical differences in the worldwide prevalence of the pandemic (Sajuthi *et al*., 2020).

## Tissue Tropism

### Studies with cell lines, tissues and organoids

Data on the tissue tropism of SARS‐CoV‐2 are important in order to understand the clinical aspects of COVID‐19. While both SARS‐CoV‐2 and SARS‐CoV (the coronavirus associated with the 2002 SARS epidemic) use the same cellular receptor ACE‐2, SARS‐CoV‐2 causes much less diarrhoea (10% of patients) than SARS‐CoV, but it has more neurological manifestations (confusion, 9%). Such distinct dissimilarity in organ tropism was also reflected in the differences in their capacity to infect pulmonary, intestinal and neurological cell lines (Chu *et al*., 2020). Ex vivo studies with human tissues obtained during surgery demonstrated that SARS‐CoV‐2 infects ciliated and mucus‐producing goblet cells and club cells in the bronchi, pneumocytes in the lung and the conjunctiva of eyes (Hui *et al*., 2020). Organoids, 3‐D cell structures grown from stem cells that recapitulate key aspects of the organs, were obtained from human small intestine and could also be successfully infected with SARS‐CoV‐2. Enterocyte progenitors were the primary viral target cells, but also differentiated enterocytes were infected and no other intestinal cell types. The viral receptor ACE‐2 was abundantly expressed on the brush border membranes of enterocytes. Viral replication occurred in intracellular ‘viral factories’ surrounded by a double membrane. Viruses were mainly released apically from the enterocytes. SARS‐CoV‐2 induced the transcription of cytokines and interferon‐stimulated genes in gut organoids (Lamers *et al*., 2020).

### Screening RNA expression databases

An impressive approach to tissue tropism was published by the US Lung Biological Network. The consortium used large single‐cell RNA sequencing (scRNAseq) data sets from primates and humans, asking which cellular subsets coexpressed ACE‐2 and TMPRSS2. Double‐positive cells are the likely support for *in vivo* viral replication. Seventeen cell types were distinguished in the lungs of rhesus monkeys, but only type II pneumocytes expressed both ACE‐2 and TMPRSS2. In lung resection material from humans, type II pneumocytes and ciliated cells showed this double expression. A striking observation both in rhesus monkey and in humans was that interferon‐induced genes were upregulated in these double‐positive cells. Absorptive enterocytes from the ileum and jejunum – both from monkeys and from the biopsies of children – coexpressed both genes, explaining the viral tropism in the gut. In the upper respiratory tract of humans, apical and ciliated cells of the ethmoid sinus and secretory goblet cells of the inferior turbinate of the nose showed this doubly positive expression pattern, and, again, showed a concomitant upregulation of an interferon alpha‐stimulated gene set. In primary human upper airway epithelial cells, the authors tested whether interferon plays an active role in upregulating ACE‐2 expression. This was indeed the case for interferon alpha, but not for interferon gamma. When screening the RNAseq database, the authors found ACE‐2 upregulation in human lung tissues infected *ex vivo* with influenza virus. These observations led to the hypothesis that coronaviruses exploit the antiviral interferon response to their advantage by increasing the expression of their cell receptor, allowing a greater number of cells to be infected. The authors caution against the use of interferon alpha in clinical trials with COVID‐19 patients (Ziegler *et al*., 2020).

## Species tropism and animal models

### Testing animals

SARS‐CoV‐2 is suspected to have originated from bats, but the intermediate host for the transfer to humans is currently unknown. Chinese scientists inoculated a number of animals with both an environmental virus isolate from the wet food market in Wuhan, from where the epidemic started, and an isolate from a patient from Wuhan early in the epidemic. Ferrets have been infected and developed an upper respiratory tract infection with fever. Outbred domestic cats have also been infected and then have developed respiratory tract symptoms and specific antibodies. Cats can transmit infection via the airborne route to other cats. Viral RNA, but not infectious virus, was detected in the intestine of infected cats. Young cats were found to be more susceptible to infection than adult cats. Dogs that have been infected showed viral RNA in the intestine, but not in the lung. No infectious virus was found in the gut of dogs, indicating low susceptibility of dogs for this virus. Pigs, chickens and ducks could not be infected with SARS‐CoV‐2 (Shi *et al*., 2020).

### Ferrets and hamsters

Animal models that reproduce the human infection are not only important for the understanding of the pathology of COVID‐19 infections but are also crucial for the preclinical testing of antiviral drugs and vaccine candidates. Ferrets, a popular model for respiratory viral infections, developed fever after intranasal inoculation with a virus from a COVID‐19 patient. Ferrets showed moderate titres of virus in the nose and low viral titres in the lung and intestine and displayed lung tissue pathology. Ferrets recovered after 2 weeks and seroconverted with neutralizing antibodies. Infected ferrets transmitted the infection efficiently in the presymptomatic stage to naïve ferrets not only via close contact, but also, albeit less efficiently, to ferrets in separate cages (Kim *et al*., 2020). Hamsters, infected with a virus from a Hong Kong COVID‐19 patient, showed weight loss, lethargy and rapid breathing. High virus titres were found in the nose, the lung and in the intestine. Histopathological changes were seen in the nose, the trachea and the lung displaying alveolar destruction, but also in the gut demonstrating epithelial necrosis. Interferon and pro‐inflammatory cytokines were rapidly induced, reached peak titres and dropped after a week with the development of neutralizing antibodies and recovery from disease. Naïve contact hamster were efficiently infected, but showed less weight loss (Chan *et al*., 2020).

### Primates

Macaques have been infected with SARS‐CoV‐2 but showed no clinical symptoms, except for moderate titres of virus excreted from the nose and throat. Maximal viral titres were seen early after infection, and titres were higher in older animals. Upon autopsy, pulmonary damage presented as the diffuse alveolar damage that is associated with replicating virus. The animals seroconverted (Rockx *et al*., 2020). Also, rhesus monkeys showed viral replication in the pharynx and severe interstitial pneumonia (Gao *et al*., 2020).

### Pangolin

Lung tissue from 17 of 25 Malayan pangolins from a wildlife rescue centre in China yielded a positive result for SARS‐CoV‐2‐related sequences. Pangolins are suspected to have been intermediate hosts at the wet food market from Wuhan, where the epidemic started. Fourteen of the pangolins died, showing lung consolidation and diffuse alveolar damage similar to human COVID‐19 victims. A complete coronavirus genome has been constructed from RNA sequencing of the lung tissues obtained from trafficked Malayan, but not Chinese, pangolins which represent two distinct species. The Malayan pangolin coronavirus is not a direct precursor to SARS‐CoV‐2, because its genome differs too much from the human isolates, but may have contributed the receptor binding domain of the spike protein gene by genetic recombination with a bat coronavirus. Pangolins and bats are both nocturnal animals that both eat insects and share overlapping ecological niches, rendering contact likely (Xiao *et al*., 2020).

### Transgenic mice

To fulfil Koch’s postulates, transgenic mice expressing the human ACE‐2 were infected with a SARS‐CoV‐2 isolate from a Chinese patient. Transgenic mice, but not wild‐type mice (which lack the viral cell receptor ACE‐2), showed symptoms of disease (interstitial pneumonia) that – partially – reproduced the disease in humans. The virus replicated in the organ where the pathology was observed, and – finally the virus could be re‐isolated from this organ. One overt clinical sign was weight loss in mice. Also, viral replication produced moderate peak titres and was limited to the lung and bronchi (Bao *et al*., 2020).

## Clinical Trials

### Chloroquine

After much media hype, political support and Emergency Use Authorization by the FDA, the initial high hopes placed in the repurposed malaria drug hydroxychloroquine administered to hospitalized COVID‐19 patients were not validated by clinical data, which had shown only mixed results. Chloroquine increases endosomal and lysosomal pH and interferes with some viral infections in cell culture. A small Chinese trial enrolling 62 COVID‐19 patients on hydroxychloroquine, or placebo, showed, in fact, a shortened period of recovery from fever and cough in the treatment group (Chen *et al*., 2020a). A French study randomizing 181 COVID‐19 patients with pneumonia on hydroxychloroquine or placebo, observed, however, no significant effect of treatment on transfer to ICU, mortality, or in the prevention of development of acute respiratory distress syndrome (Mahévas *et al*., 2020). Eighty‐one adult Brazilian patients with severe COVID‐19 were randomized on a high or a low dose of chloroquine plus azithromycin and oseltamivir. A placebo group was considered to have been unethical due to media support for chloroquine use. Overall mortality was 27%, which was not lower than in historical controls. Mortality was substantially higher in the high‐dose group compared with the low‐dose chloroquine group (39 vs. 15%), and the high‐dose regime was stopped in this trial by the data safety monitoring board. On day 4 of hospitalization, more than 80% of the patients in both groups showed viral RNA in nasopharyngeal secretions excluding an antiviral effect (Borba *et al*., 2020). One hundred and fifty COVID‐19 patients from China were treated with oral hydroxychloroquine (HCQ), and this was compared with standard treatment alone in an open clinical trial. The treatment did not result in an accelerated clearance of viral RNA in upper and lower respiratory tract secretions. Clinical symptom alleviation within 28 days was not different between the two groups. A more rapid symptom alleviation was seen for HCQ, over the control group during the second week of treatment, but it was not statistically significant. This trend became even more visible when in a post hoc analysis only cases of patients without other antiviral agent treatment were investigated. HCQ led to a more rapid normalization of C‐reactive protein levels compared with those in the controls. HCQ addition led to a higher rate of adverse events over the controls (30% vs 9%), which consisted mostly of diarrhoea (Tang *et al*., 2020b).

From 1376 consecutive COVID‐19 patients hospitalized in New York City, 811 were treated with a high dose of HCQ on day 1, and a low dose during the following 4 days. Half of them received the high dose within 24 h of hospitalization, and the follow‐up course was for three weeks. There were 565 COVID‐19 patients who did not receive HCQ. The primary end‐point was intubation or death, which occurred in 25% of the patients. In crude, unadjusted analysis of this observational study, HCQ‐treated patients were 2.4 times more likely to reach the detrimental primary end‐point than non‐treated patients. Since HCQ‐treated patients were, at baseline, more severely ill than non‐treated patients (by oxygenation index), the 811 cases of HCQ patients were compared in a second analysis to those of 274 better matched non‐treated patients. Neither benefit nor harm has been associated with HCQ use (Geleris *et al*., 2020).

### Remdesivir

In an open‐label and compassionate trial, 53 patients with severe COVID‐19, in which 34 of them were on invasive ventilation and received remdesivir, a nucleotide analogue developed against Ebola virus. After 3 weeks of follow‐up, 68% of them showed clinical improvement; 57% of patients on mechanical ventilation were extubated, while 13 % died. This fatality rate is a relatively better result when compared with a 66% mortality rate in Chinese patients receiving invasive ventilation. Due to a small study size, short length of follow‐up and the lack of a randomized control group, the outcome must be interpreted with caution (Grein *et al*., 2020).

In Hubei, there were 237 Chinese patients with severe COVID‐19 who were subsequently randomized on a 2:1 basis for intravenous remdesivir, or placebo, in a multicentre clinical trial. The time it took to show clinical improvement in the remdesivir group was statistically not significantly different from that of the control group (median 21 vs. 23 days). The time from onset of symptoms to start of treatment was 10 days. Patients receiving the treatment during the first 9 days showed a numerically greater difference for clinical improvement (18 vs. 23 days), but this difference is not statistically significant. The length of time of viral excretion in upper and lower respiratory tract samples did not differ between the two groups, demonstrating a lack of *in vivo* antiviral activity of this nucleoside analog. The mortality rate was 15% in the remdesivir group, and 13% in the placebo group. This otherwise well‐conducted clinical trial was underpowered because it could not attain the enrolment of the planned 453 patients, since the epidemic stopped in Hubei as a consequence of containment measures. Both groups did not differ in the types of, or number of adverse events, demonstrating the safety of intravenous remdesivir (Wang *et al*., 2020).

On 1 May, the FDA granted an emergency use authorization for remdesivir to physicians. The decision was based on the outcome of a clinical trial, which showed an accelerated recovery in more than 1000 enrolled patients treated with either remdesivir or placebo (11 vs. 15 days). There were fewer deaths in the group with treatment than in the placebo group, but the difference was not statistically significant. Details of the trial have not yet been published (Ledford, 2020).

### Lopinavir and arbidol

A total of 86 COVID‐19 cases of patients from China with mild/moderate disease were randomized on the antiviral lopinavir (an inhibitor of HIV protease combined with ritonavir, which prolongs the presence of drugs in the body) or the antiviral arbidol (an influenza virus fusion inhibitor only registered in Russia) or in a control group in a 2:2:1 ratio. The primary end‐point was positive‐to‐negative conversion for viral RNA in pharyngeal swabs. No difference was seen between the groups. Secondary outcomes were represented by clinical parameters: fever, cough and lung CT improvement. Again, no difference was seen between the groups. Adverse events were not observed in the controls, but in 35% and 14% of the lopinavir and arbidol group, respectively, mainly manifested as diarrhoea. Thirteen patients progressed to severe disease during hospitalization: namely 8, 3 and 2 in lopinavir, arbidol and control group, respectively. The study is small and was only conducted at a single centre, but it still confirms the lack of efficacy of lopinavir/ritonavir treatment in severe COVID‐19 cases (Li *et al*., 2020b).

In a retrospective cohort study with 33 Chinese patients suffering from moderate COVID‐19 showing lung CT abnormalities, 16 patients were treated with an arbidol/lopinavir/ritonavir combination while 17 patients received only lopinavir/ritonavir. The group including arbidol in the combination showed a significantly shorter nasopharyngeal and stool excretion of SARS‐CoV‐2, and an accelerated normalization of lung CT, compared with the control group. The positive outcome must, however, be interpreted with caution since the two groups differed significantly for glucocorticoid treatment (6% vs. 41% in control). The better outcome in the arbidol arm could therefore be due to less use of detrimental glucocorticoid (Deng *et al*., 2020).

### Triple therapy: lopinavir, ribavirin, interferon beta‐1b

In a multicentre trial, 127 COVID‐19 patients from Hong Kong were enrolled into a prospective, randomized, open‐label phase 2 clinical trial. Eighty‐six patients received a combination treatment of the lopinavir (plus ritonavir), the nucleoside analog ribavirin and interferon beta‐1b, while the control group was only treated with lopinavir (plus ritonavir). At baseline, both groups were well matched and showed mild‐to‐moderate disease. The combination group demonstrated, in comparison with the controls, a significantly reduced time (7 vs. 12 days) to clearance of viral RNA in the nasopharynx (the primary end‐point), as well as a shortening of oropharynx, throat and stool virus excretion. The combination therapy was also associated with significant clinical improvement compared with controls, and a shorter hospitalization stay. There were no fatalities. The treatment group also displayed lower IL‐6 values than the controls. A post hoc subgroup analysis comparing 76 patients (52 in combination and 24 in control group), who started treatment less than 7 days after symptom onset, showed again a significant difference between the two groups. In contrast, no difference was seen between the two groups when the treatment was initiated after 7 days. Out of concern for safety, patients in the combination group did not receive interferon at later stages of the infection. The therapeutic effect in the combination group could be attributed to either earlier ribavirin treatment or interferon inclusion (Hung *et al*., 2020).

### IL‐1 receptor antagonist

Anakinra is a recombinant interleukin IL‐1 receptor antagonist. High‐dose intravenous anakinra has FDA and EMA off‐label approval for the treatment of hyperinflammatory conditions such as macrophage activation syndrome (see above coagulopathy). A Chinese study used anakinra in severe COVID‐19 patients who could not receive intensive care due to overwhelming patient numbers. Low‐dose subcutaneous anakinra (the standard application) had no effect on C‐reactive protein levels or clinical parameters, compared with controls. High‐dose intravenous anakinra application suppressed hyperinflammation, as documented by a decrease in C‐reactive protein, and resulted in a significantly higher rate of survival at 3 weeks compared with controls (90 vs. 56%). The retrospective nature and the small number of patients treated limit positive conclusions (Cavalli *et al*., 2020).

### Anti‐IL‐6R monoclonal antibody

According to a popular pathogenesis model, interleukin IL‐6 production induces immunopathology in severe COVID‐19 cases. Tocilizumab, a recombinant humanized monoclonal antibody, which binds the IL‐6 receptor and inhibits its signal transduction, has been previously used in the treatment of a special form of juvenile arthritis associated with increased IL‐6 production. This monoclonal antibody led to a rapid increase in blood lymphocytes in six COVID‐19 patients (Giamarellos‐Bourboulis *et al*., 2020). Clinicians from China gave tocilizumab to 21 patients with severe or critical COVID‐19 who had failed to respond to standard therapy. One day after the injection of tocilizumab, the fever disappeared in all patients, C‐reactive protein returned to normal in most of them, peripheral oxygen saturation improved, CT‐documented lung damage ameliorated, and the virus was cleared. Adverse effects were not observed, and all patients were discharged (Xua *et al*., 2020).

### Plasma

Three weeks after the onset of disease, 6 severely ill COVID‐19 patients received plasma from convalescent COVID‐19 patients, while 11 COVID‐19 patients received non‐convalescent plasma as a control. Convalescent plasma application was linked to a significantly higher viral clearance over controls (100 vs 21%) but did not affect mortality which was high in both groups (5/6 and 10/11 died) (Zeng *et al*., 2020).

### Heparin

In a retrospective study from China, 20% of 449 severely ill COVID‐19 patients were treated with the coagulation inhibitor heparin. Mortality was 30%, irrespective of whether heparin was given or not. However, when patients were stratified with respect to coagulopathy at baseline (using D‐dimer concentration sixfold higher than normal as threshold), a 20% reduction in mortality was associated with heparin use. The data need to be interpreted with caution, since they come from a retrospective subgroup analysis (Tang *et al*., 2020a).

## Towards new antivirals

### Hydroxycytidine

A promising prodrug is the nucleotide analog hydroxycytidine. In cell culture infections, this drug is active against a wide range of human and bat coronaviruses thus potentially offering inhibitory activity against future spill‐over infections from animals (zoonosis). The drug increases the mutation rate of the virus during replication resulting in infectivity losses. In mouse infection models for SARS‐CoV and MERS virus, it had good oral bioavailability, ameliorated lung function, reduced virus titre and diminished weight loss caused by the viral infection. The drug demonstrated therapeutic effects up to 24 h after infection, which is substantial since mice show a compressed disease course compared to that of humans. Coronaviruses that had become resistant to remdesivir showed enhanced susceptibility to inhibition by hydroxycytidine (Sheahan *et al*., 2020). Hydroxycytidine causes mutagenesis in the bacterium *Escherichia coli* and the fungus *Neurospora crassa*.

### Cell protease inhibitor

SARS‐CoV‐2 needs, after the docking of its spike protein to the cell receptor ACE‐2, a proteolytic cleavage of the spike protein at a polybasic site separating the S protein into two protein fragments S1 and S2, where S2 mediates the fusion of the viral and cell membranes, which leads to the entry of the viral genome into the infected cell. Cell culture infection tests demonstrated that SARS‐CoV‐2 uses two proteases for this proteolytic processing, either the lysosomal cathepsin CatB/L or the transmembrane protease TMPRSS2. The serine protease inhibitor camostat, which is a registered drug in Japan for gastroenterology problems, inhibits TMPRSS2 and confers partial resistance to infection with SARS‐CoV‐2, and total protection when combined with E‐64d, an inhibitor of CatB/L (Hoffmann *et al*., 2020).

### Viral protease inhibitor

Chinese scientists have targeted the SARS‐CoV‐2 main protease for inhibition. After expressing the protease and designing a fluorescence‐labelled substrate, they used, as an inhibitor, compound N3 which was active against SARS‐CoV. In silico docking verified that it could fit into the predicted structure of SARS‐CoV‐2 main protease. Kinetic analysis revealed a two‐step inactivation process resulting in N3 covalent binding to the catalytic site. The crystal structure for the protease‐bound N3 was solved and was followed by a virtual screening of a chemical database for better inhibitors. The next step was a high‐throughput screening of 10’000 compounds. One of the best inhibitors was an organo‐selenium compound that inhibited the infectivity of SARS‐CoV‐2 in a cell culture test. Since this compound has already been investigated for the treatment of several diseases, where it showed a high safety profile, this repurposed drug could be re‐entered into clinical trials relatively quickly (Jin *et al*., 2020).

### Chemical database screening

US researchers have expressed in a cell culture system all of the SARS‐CoV‐2 proteins that were tagged with a recognition peptide, which allowed the isolation of cellular proteins interacting with the viral bait protein. The protein complexes were collected by affinity chromatography, and the copurified cellular proteins were identified by mass spectrometry. This approach yielded 332 high‐confidence protein–protein interaction partners. The captured proteins showed high expression in lung tissue, and many of them also interacted with another lung pathogen, *Mycobacterium tuberculosis*. The viral proteins interacted with proteins from multiple innate immune pathways, the host translation machinery, the ubiquitin ligase complex and proteins involved in transcriptional regulation of antiviral responses. From a search of chemical databases, the scientists identified 69 ligands, including FDA‐approved drugs and compounds currently used in clinical trials that interacted with the identified proteins. In cell culture, several compounds showed inhibitory activity against SARS‐CoV‐2. The candidate inhibitors included the following: antihistamines, antitussives, antipsychotics and interestingly also hydroxychloroquine (Gordon *et al*., 2020).

### Traditional Chinese Medicine (TCM)

Parallel to these state‐of‐the‐art biochemical approaches, one must also mention the traditional approaches promoted in China. According to Chinese state media, Jinhua Qinggan herbal granules accelerated, in a clinical trial with COVID‐19 patients, conversion to a negative virus test within 2 days. Xuebijing, an extract of five herbs, reduced mortality in COVID‐19 by 8.8% via removing blood stasis. Details of these trials are not yet available (Cyranosky, 2020).

### Hypertension medication and Covid‐19

In the emergency situation of the expanding pandemic and in an effort to save lives, severe COVID‐19 cases were frequently treated with many different drugs. This situation leads to complex drug interaction, potential detrimental side effects for the patients and difficulty to disentangle the effect of tested drugs in clinical trials from concomitant treatment. The extent of the pandemic also led to questions about whether the drug treatment for existing morbidities of the patients increased the risk for SARS‐CoV‐2 infections, or even led to a more severe clinical course. From the large selection of literature about treatment of other diseases during the COVID‐19 epidemic, I have selected one which was cited as a major risk for developing severe COVID‐19, namely hypertension.

ACE‐2 is the cellular receptor of SARS‐CoV‐2, and it is the target for a class of antihypertensive drugs that block this enzyme, which leads to its increased expression. Since hypertension has been frequently reported as a risk factor in severe COVID‐19 infections, the safety of antihypertensive drugs during the COVID‐19 epidemic has been questioned. Cardiologists studied this question in New York City, an epicentre of this pandemic. More than 12’000 cases were investigated, nearly half of them had a positive viral test. From these 6’000 cases, 1’000 had severe COVID‐19, 726 were in intensive care, and 447 died. The researchers ruled out a significant effect from any of five common antihypertensive drugs favouring infection (i.e. from being tested positive for SARS‐CoV‐2) or that they increase the likelihood of inducing severe COVID‐19. Patients taking beta‐blockers even had a significant, although marginally lower, risk of testing positive for the virus (Reynolds *et al*., 2020).

The same question was addressed in another epicentre of the pandemic, in Lombardy, with a large case–control study involving 6’272 COVID‐19 patients and 30’000 controls, matched for age, sex and municipality. Angiotensin II receptor blockers (ARB) and ACE inhibitors (ACEI), common antihypertensive drugs, were more frequently used by COVID‐19 patients than in the control group (ARB: 22 vs 19%; ACEI: 24 vs. 21%). After multivariate analysis, neither ARB, ACEI nor other common antihypertension drugs had a significant association with COVID‐19. The Italian study confirmed the Chinese observations that the COVID‐19 patients were in poorer health than the general population matched for age, particularly with respect to previous hospitalization for cardiovascular diseases (Mancia *et al*., 2020).

These conclusions were corroborated in another study in which 8910 hospitalized COVID‐19 patients were compared for outcome, namely death (*n* = 515) versus survival to discharge (*n* = 8395 patients). The cases were from hospitals in North America, Europe and Asia. In multivariate analysis: age greater than 65 y, coronary artery disease, congestive heart failure, cardiac arrhythmia, chronic obstructive pulmonary disease and current smoking were all associated with a higher risk of in‐hospital death. ACE inhibitor use was associated with a significantly lower risk of death, but might be a coincidental observation for multiple comparisons. However, the data do not provide evidence for a detrimental effect of antihypertensive drugs on COVID‐19 mortality (Mehra *et al*., 2020).

## Conclusion

Until now no prospective, sufficiently powered, controlled clinical trial that has been reported in a peer‐reviewed publication has reported an impact of drug interventions on COVID‐19 mortality, despite more than 1’000 trials registered on ClinicalTrials.gov, including 600 intervention studies. Technical problems, such as the lack of true control groups, concomitant other treatments, make it difficult to disentangle the effects. Many trials were started before the insight into COVID‐19 pathogenesis, which are now accumulating, became available. Probably, reliable biomarkers for different pathological types of COVID‐19 forms are needed in order to allow more precisely targeted therapeutic interventions (Bauchner and Fontanarosa, 2020). Many approaches are with repurposed drugs, developed for other infections, in order to cut down the time needed for drugs to reach patients in an emergency situation. These approaches need to be complemented by trials specifically tailored to the COVID‐19 pathology. A. Fauci reminded us that drug trials of HIV infections progressed in small steps before effective drug combinations could be developed.

## Conflict of interest

The author consults Nestlé, his former employer, on the scientific aspects of the COVID‐19 epidemic, but he does not consider this as a conflict of interest.
